# Efficacy and safety of subthreshold micropulse laser for chronic central serous chorioretinopathy: a systematic review and meta-analysis

**DOI:** 10.3389/fmed.2026.1785787

**Published:** 2026-03-13

**Authors:** Wei Luo, Yongning He

**Affiliations:** Department of Ocular Fundus Diseases, Nanjing Aier Eye Hospital, Nanjing City, Jiangsu, China

**Keywords:** chronic central serous chorioretinopathy, efficacy, meta-analysis, safety, subthreshold micropulse laser therapy

## Abstract

**Background:**

Chronic central serous chorioretinopathy (cCSC) can lead to irreversible visual impairment. Traditional treatment methods are limited by their efficacy and the risk of complications. Subthreshold micropulse laser (SML) therapy has emerged as a promising treatment option.

**Objective:**

This study systematically evaluated the efficacy and safety of SML for treating cCSC, providing evidence-based clinical recommendations.

**Methods:**

We searched PubMed, Embase, Cochrane Library, and Web of Science for studies published up to January 10, 2025, evaluating SML for cCSC. Eligible designs included randomized controlled trials (RCTs), prospective cohort studies, and case series. Two researchers independently performed literature screening, data extraction, and quality assessment using RoB 2, NOS, and JBI tools, respectively. The certainty of evidence was evaluated using the GRADE system. Meta-analysis was conducted using STATA 18.0 to pool the effects on visual acuity (BCVA) and retinal morphology (CMT, CT, CRT). Subgroup analyses were performed based on laser wavelengths (527, 532, 577, and 810 nm).

**Results:**

A total of 1,412 articles were identified, and 21 studies were included, with a total sample size of 939 eyes. Meta-analysis demonstrated that SML significantly improved the best-corrected visual acuity (BCVA) of cCSC patients, with a pooled effect size of [SMD = −0.74, 95% CI (−1.14, −0.33), *P* = 0.001]. The efficacy was consistent across different wavelengths (527, 532, 577, 810 nm). Regarding retinal morphological parameters, SML significantly reduced the central macular thickness [CMT, SMD = −2.15; 95% CI (−3.82, −0.47), *P* = 0.018], choroidal thickness [CT, SMD = −0.43; 95% CI (−0.79, −0.08), *P* = 0.025], and central retinal thickness [CRT, SMD = −1.12; 95% CI (−1.34, −0.90), *P* = 0.001]. Although improvements in subretinal fluid height (SRFH) were not statistical significance, most studies indicated a positive trend. Additionally, SML demonstrated a high safety profile for treating cCSC.

**Conclusion:**

SML significantly improves visual function and retinal morphology in cCSC patients, demonstrating consistent efficacy across different wavelengths and providing a safe, effective treatment option for cCSC.

## 1 Introduction

Central serous chorioretinopathy (CSC) is a retinal disorder characterized by serous retinal detachment, often accompanied by focal or multifocal changes in the retinal pigment epithelium (RPE). This disease primarily affects middle-aged men, and its pathogenesis is closely related to factors such as elevated corticosteroid levels and high-stress states (1). Based on the disease course, CSC is generally classified into acute and chronic types (2). Of the acute CSC cases, 80–90% typically resolve spontaneously within 3–6 months (3). However, approximately 20–30% of patients will experience one or more recurrences of acute CSC, and about 5% may progress to chronic central serous chorioretinopathy (cCSC) (4). Chronic CSC is characterized by the prolonged presence of subretinal fluid, leading to retinal thinning and irreversible photoreceptor damage, ultimately causing severe visual impairment and significantly impairing patient’s quality of life.

At present, the treatment of cCSC remains a significant challenge in the field of ophthalmology. Clinically, a variety of treatment methods are commonly used, including drug therapy (corticosteroid antagonists, anti-vascular endothelial growth factor drugs), traditional laser therapy, and photodynamic therapy (PDT) (5, 6). However, these approaches generally suffer from issues such as large variations in efficacy, high risks of complications, and heterogeneity in research findings (7), which has led to the absence of a unified treatment consensus. Therefore, developing a treatment plan that combines safety with high efficacy is of great clinical importance.

Subthreshold micropulse laser (SML) therapy is an emerging laser treatment technique. By dividing the laser energy into short pulses, it significantly reduces the risk of thermal damage to the retina while promoting the repair of RPE (8). This therapy has been widely applied in various clinical studies for the treatment of retinal diseases and has been approved by the U.S. Food and Drug Administration (9), suggesting its potential as an alternative treatment for CSC. Studies have shown that SML exhibits promising efficacy and safety in conditions such as diabetic macular edema and macular edema secondary to retinal vein occlusion (10, 11). In the treatment of CSC, SML achieves precise therapy by selectively targeting RPE cells. Beyond the photothermal interaction with melanosomes, the biological effects of SML are believed to rely mainly on sublethal, repetitive thermal stimulation of retinal pigment epithelium cells, rather than on visible coagulative damage (1–3). This subthreshold stimulation can activate intracellular stress-response pathways and enhance RPE metabolic and pump functions, including fluid transport and barrier regulation. Experimental and clinical studies suggest that SML may upregulate heat shock proteins and modulate cytokine and growth factor expression, thereby improving RPE cell viability and functional recovery without inducing retinal scarring (4–6). Targeting RPE cells represents a rational therapeutic strategy in cCSC because RPE dysfunction is a central event in disease pathophysiology (7, 8). In cCSC, impaired RPE barrier integrity and reduced fluid pumping capacity contribute directly to persistent subretinal fluid accumulation. By functionally stimulating RPE cells, SML is thought to promote resorption of subretinal fluid, restore outer blood–retinal barrier function, and normalize the metabolic interaction between the RPE and photoreceptors. Therefore, the effectiveness of SML in cCSC is not limited to selective melanosome absorption but also involves biological activation and functional restoration of RPE, which helps explain its clinical benefits in improving retinal morphology and visual outcomes while maintaining a favorable safety profile (1, 9). The mechanism involves generating high peak temperatures around melanosomes within RPE cells, rupturing cell membrane without damaging adjacent photoreceptor cells and preventing scar formation (7). Compared to traditional laser therapies, SML enables more precise targeting of treatment areas, significantly reducing the risk of RPE damage and visual impairment. Notably, the PLACE trial demonstrated that half-dose PDT significantly improved retinal anatomy and sensitivity compared to SML treatment alone (12), but PDT carries multiple clinical risks and therapeutic limitations (13).

Due to its unique mechanism and clinical advantages, SML therapy is considered promising with broad potential and expected to become an effective alternative to existing CSC treatments. However, preliminary studies exploring SML therapy for cCSC suffer from small sample sizes, varying methodological quality, and a lack of systematic reviews or meta-analyses. As a result, considerable uncertainty regarding on the efficacy and safety of SML therapy for cCSC. Therefore, this study aims to comprehensively evaluate the clinical effects and safety of SML therapy through a systematic review and meta-analysis, providing reliable evidence for clinical application. It focuses on efficacy indicators including visual improvement and retinal thickness changes, along with rigorous safety data. This research seeks to validate the clinical value of SML therapy, potential advantages and limitations for cCSC treatment. Ultimately, this study provides new perspectives for treatment strategies and promote standardized clinical practice of SML therapy.

## 2 Methods

### 2.1 Literature search strategy

We conducted a comprehensive literature search across several English-language databases, including PubMed, Web of Science, Embase, and Cochrane Library, for articles published up to January 10, 2025. The search terms used were “Chronic Central Serous Chorioretinopathy,” “Micropulse Laser Therapy,” “Treatment Outcome,” or “Safety,” utilizing a combination of subject headings and free terms (Supplementary table 1).

Following the literature search, one researcher removed duplicate records. Titles and abstracts of the remaining records were screened independently by two reviewers (WL and YH). Full texts of the potentially relevant studies were then retrieved and further assessed independently by the same two reviewers (WL and YH). Any disagreements regarding study eligibility were resolved through discussion, and when necessary, by consultation with the principal investigator. Additionally, reference lists of included studies were checked to identify any potentially missed relevant research.

### 2.2 Inclusion and exclusion criteria

### 2.2.1 Inclusion criteria

(1) Study Population: Patients with a clear diagnosis of cCSC, characterized by sub-retinal fluid (SRF) persisting ≥ 3 months, diffuse retinal pigment epithelium (RPE) pathology, and absence of acute features or spontaneous resolution tendency; (2) Intervention: Intervention group treated with SML therapy; (3) Control Group: No intervention or treatment with other laser therapies; (4) Outcome Measures: Primary efficacy outcomes include best-corrected visual acuity improvement, retinal morphology, etc. Safety evaluation includes reporting treatment-related adverse events and their incidence; (5) Study Design: Interventional studies, including randomized controlled trials (RCTs), prospective cohort studies, and case series (single-arm studies).

#### 2.2.2 Exclusion criteria

(1) Non-cCSC patients or those not definitively diagnosed with cCSC; (2) non-clinical studies or research studies not focusing on therapeutic interventions; (3) basic research, case reports, conference abstracts, letters to editors, commentaries/editorials, reviews, or discussion drafts; (4) study protocols; (5) studies with incomplete data or unclear methodologies; (6) articles published in languages that cannot be translated; (7) duplicate studies published in other literature.

### 2.3 Literature screening

(1) Initial Screening: Based on title and abstract, studies were screened to exclude non-clinical research, basic research, studies unrelated to SML methods, or those not reporting the primary outcome measures. (2) Secondary Screening: Full-text screening was performed to assess the studies according to the inclusion and exclusion criteria. The quality of the studies, such as research design, sample size, data collection, and analysis methods, was evaluated. (3) Recording Screening Results: The results of the screening, including included and excluded studies and reasons for exclusion, were documented.

### 2.4 Data extraction

Two reviewers (WL and YH) independently extracted relevant data from the included studies. The extracted information included: the first author, publication year, sample size, country, average age, micropulse intervention wavelength and parameters, visual acuity improvement, retinal morphology, and other efficacy outcomes, treatment-related adverse events, and associated outcome measures (mean values, standard deviations). A standardized data extraction form was used to record and verify the data to ensure accuracy and completeness.

### 2.5 Quality assessment

The included studies comprised three main designs: randomized controlled trials (RCTs), prospective cohort studies, and case series (single-arm studies without a concurrent control group). The methodological quality of each study type was assessed using appropriate tools. For RCTs, the revised Cochrane Risk of Bias Tool (RoB 2) was applied, which evaluates bias across five domains: randomization process, deviations from intended interventions, missing outcome data, measurement of the outcome, and selection of the reported result. Each domain was judged as “low risk,” “some concerns,” or “high risk,” and an overall risk of bias judgment was derived. For prospective cohort studies, the Newcastle-Ottawa Scale (NOS) was used. The NOS assesses studies on three domains: Selection (maximum 4 points), Comparability (maximum 2 points), and Outcome (maximum 3 points), with a total score of 9. Studies scoring 7–9, 5–6, and ≤ 4 were considered high, moderate, and low quality, respectively. For case series studies, the Joanna Briggs Institute (JBI) Critical Appraisal Checklist for Case Series was employed. This checklist consists of 10 items evaluating aspects such as clear case definition, completeness of reporting, and reliability of measurement. Each item is answered as “Yes,” “No,” “Unclear,” or “Not Applicable.” Two investigators (WL and YH) independently performed all quality assessments. Any discrepancies were resolved through discussion or consultation with a third reviewer.

The quality of the outcome measures was evaluated using the GRADE (Grading of Recommendations, Assessment, Development, and Evaluation) system. Based on factors such as risk of bias, inconsistency, indirectness, imprecision, and publication bias, the evidence quality was rated as high, moderate, low, or very low.

### 2.6 Statistical analysis

Meta-analysis was conducted using STATA 18.0 software (STATA Corp., College Station, TX, United States). Mean differences (MD) or standardized mean differences (SMD) with their 95% Confidence Intervals (CIs) were extracted from the included studies. Heterogeneity between studies was assessed using the I^2^ statistic, with values ranging from 0 to 100%. Depending on the *I*^2^ value, a random-effects or fixed-effects model was used to pool the data. To assess publication bias, Begg’s test and Egger’s test were performed, and a funnel plot was generated to visually assess the potential for publication bias. Egger’s test was used to quantify the symmetry of the funnel plot, with a *p* < 0.05 considered statistically significant for publication bias. Sensitivity analysis was conducted to verify the robustness of the pooled results and ensure the reliability of the study’s conclusions. Subgroup Analysis: Subgroup analyses will be performed to evaluate the efficacy differences based on varying laser wavelengths and other factors.

## 3 Results

### 3.1 Study selection and characteristics

**FIGURE 1:**
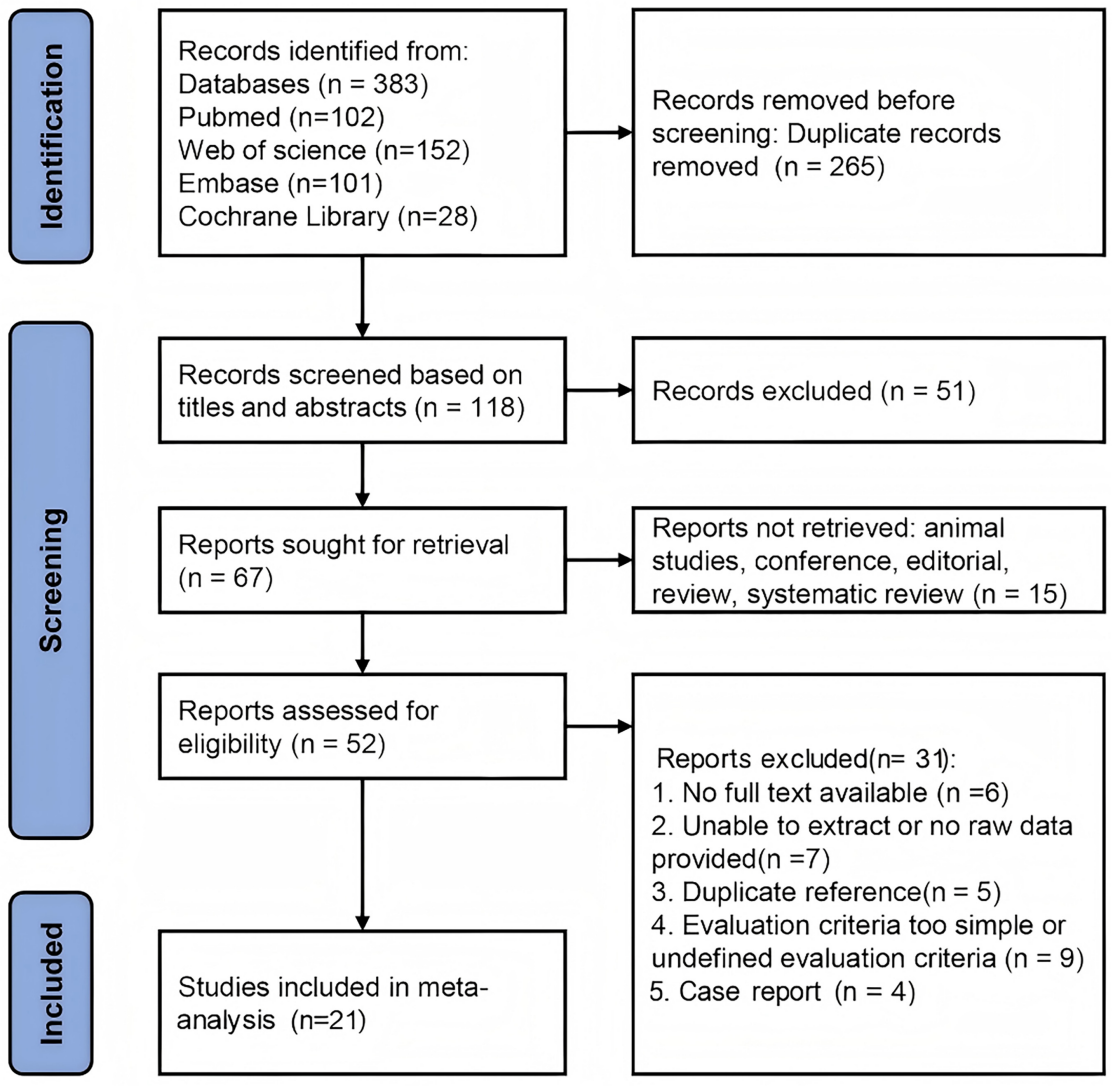
Literature search process summary based on the preferred reporting items for systematic reviews and meta-analyses (PRISMA) guidelines.

A literature search was conducted across databases including PubMed, Web of Science, Embase, and Cochrane Library for articles published up to January 10, 2025. A total of 1,412 studies were initially identified. After removing duplicates, 910 articles remained. Based on title and abstract screening, 727 studies were excluded for not meeting the thematic criteria, leaving 183 studies for full-text evaluation. Of these, 131 studies were excluded due to not meeting the inclusion criteria, such as animal experiments, conference abstracts, editorials, reviews, or systematic reviews. This left 52 articles for further screening. Upon full-text review, 30 studies were excluded for the following reasons: 7 lacked full text, 7 did not provide original or extractable data, 5 were duplicates, 9 had overly simplistic or undefined evaluation criteria, and 3 were case reports. In total, 21 articles were included in the final Meta-analysis. A flow diagram illustrating the literature search and study selection process is shown in Figure 1.

### 3.2 Study characteristics

A total of 21 studies were included in this Meta-analysis, comprising 916 patients diagnosed with cCSC, with a total of 939 eyes included in the analysis. The mean age of participants ranged approximately from 36 to 55 years. All patients included in the studies were diagnosed with varying degrees of cCSC, and all intervention groups received SML treatment. The study designs included 4 randomized controlled trials, 5 prospective cohort studies, and 12 case series. The studies were conducted in several countries and regions, including China, the United States, India, Spain, Turkey, and France, reflecting the clinical practice variations worldwide. All studies were carried out at hospital ophthalmology centers or retinal specialty departments, ensuring the professionalism of diagnosis and treatment as well as the reliability of the data. A summary of the demographic data and intervention details of the included studies is shown in Table 1.

**TABLE 1:** Summary of demographic data and intervention details of included studies.

Study	Year	Country	Study Type	Number of patients	Number of eyes	Age (years)	Wavelength	Intervention parameters	Efficacy indicators	Adverse effects
Ambiya et al. (27)	2024	India	RCT	198	99/99	36.48 ± 5.23	810 nm; 532 nm	Spot size of 100 μm, exposure time of 0.2 s, duty cycle of 5%	BCVA, CMT	None
Torrellas et al. (14)	2023	Spain	Prospective cohort study	37	43	55 ± 10.2	577 nm	Spot size of 160 μm, 5% duty cycle	BCVA, CT	None
Sun et al. (15)	2020	China	RCT	88	88	44.41 ± 8.71; 44.68 ± 6.77	577 nm	Spot size of 160 μm, duty cycle of 5%	BCVA, CRT	12% mild RPE depigmentation in SML group
Uzlu et al. (16)	2021	Turkey	Prospective cohort study	19	20	48.9 ± 9.40	577 nm	Spot size of 100 μm, exposure time of 0.2 s, 160–200 mW	BCVA, CRT, ONL	None
Işık et al. (17)	2020	Turkey	Case series	58	58	42.4 ± 9.9	577 nm	Spot size of 160 μm, exposure time of 0.2 s,duty cycle of 5%	CMT, SRFH	-
Kayhan et al. (18)	2022	Turkey	Case series	16	18	47.3 ± 5.7	577 nm	Spot size of 200 μm	BCVA, CMT, CT, SRFH	None
Liu et al. (19)	2024	China	Case series	31	33	51.72	577 nm	Spot size of 160 μm, exposure time of 0.2 s	ONL	None
Oribio-Quinto et al. (20)	2024	Spain	Prospective cohort study	40	42	54.5 ± 11.5	577 nm	Spot size of 150 μm, exposure time of 0.2 s, duty cycle of 5%	BCVA, CT, SRFH	1 patient with significant loss of 15 letters due to progressive ORL and RPE atrophy
Gawêcki et al. (21)	2017	Poland	Case series	51	51	53.8 ± 11.2	577 nm	Spot size of 160 μm, exposure time of 0.2 s, duty cycle of 5%, 250 mW	BCVA, CRT, SRFH	None
Roisman et al. (28)	2013	Brazil	RCT	15	15	39.5 ± 7.7	810 nm	Spot size of 125 mm,a continuous wave mode and 300-ms duration, duty cycle of 15%, the power was increased by 1.2 × threshold.	BCVA, CMT	
Amoroso et al. (22)	2022	France	Prospective cohort study	36	39	51.87 ± 10.4	577 nm	exposure time of 0.1 s, 315.23 mV, spot number of 435.89	BCVA, CMT, CT	-
Lanzetta et al. (29)	2008	Italy	Case series	22	24	47 ± 9	810 nm	Spot number of 215,duty cycle of 15%	CRT	None
Ambiya et al. (30)	2020	India	Prospective cohort study	21	23	37.09 ± 3.27	532 nm	Spot size of 100 μm, exposure time of 0.2 s, duty cycle of 5%	BCVA, CRT	None
Kim et al. (23)	2019	Korea	Case series	27	27	45.1 ± 8.6	577 nm	Spot size of 100 μm, exposure time of 0.02 s, duty cycle of 15%, 200–400 mW	BCVA, CMT	None
Kim, et al. (24)	2015	Korea	Case series	10	10	43.9 ± 6.24	577 nm	Spot size of 100 μm, exposure time of 0.02 s, duty cycle of 15%	BCVA, CMT, CT	None
Maruko et al. (25)	2017	Japan	Case series	28	29	43 ± 12	577 nm	Spot size of 200 μm, exposure time of 0.2 s, 60–80 mW	CT	1 patient had RPE damage
Kustryn et al. (26)	2024	Ukraine	Case series	30	30	43 ± 14	577 nm	Spot size of 100 μm, exposure time of 0.2 s, and duty cycle of 9%	BCVA, CRT, SRFH	5 patients had localized RPE scarring
Scholz et al. (35)	2015	Netherlands	Case series	38	38	51 ± 18.5	577 nm	Spot size of 160 μm, exposure time of 0.2 s, duty cycle of 5%	BCVA, CRT	None
Yadav et al. (36)	2015	India	Case series	13	15	49 ± 11	577 nm	Spot size of 100 μm, exposure time of 0.2 s, duty cycle of 10%	BCVA	None
Arsan et al. (37)	2018	Turkey	Case series	39	39	43.38 ± 13.85	577 nm	Spot size of 160 μm, exposure time of 0.02 s, duty cycle of 5%	BCVA	None
Oh et al. (31)	2021	Korea	RCT	68	68	45.6 ± 7.0	527 nm	Pulse duration 1.7 μs, repetition rate of 100 Hz, pulse energy of 30 to 350 μJ	BCVA, CMT, SRFH	Reported 5 urgent ocular adverse events

BCVA, Best Corrected Visual Acuity; CMT, Central Macular Thickness; CT, Choroidal Thickness; CRT, Central Retinal Thickness; SRFH, Height of Subretinal Fluid; ONL, Outer Nuclear Layer.

In the included studies, there was a noticeable distribution in the wavelength parameters used for micropulse laser treatment. Specifically, the 577 nm wavelength was the most widely used, with 17 studies employing this wavelength (14–26); The 810 nm wavelength was used in 3 studies (27–29); while 532 nm and 527 nm wavelengths were applied less frequently, in 2 studies (27, 30) and 1 study (31), respectively. In terms of intervention parameters, the settings were relatively consistent across the studies: the spot size was mostly set between 100 and 200 μm, with exposure times ranging from 0.02 to 0.2 s, and the duty cycle controlled between 5 and 15% (Table 1). These settings aimed to balance treatment efficacy with tissue safety, providing important technical references for micropulse laser therapy.

### 3.3 Quality assessment

The methodological quality assessment results for the included studies are detailed in Tables 2–4. Among the four RCTs assessed using the Cochrane RoB 2 tool (Table 2), one study was judged to have an overall “low risk” of bias, while three raised “some concerns,” primarily in the domains of randomization process or measurement of the outcome. For the five prospective cohort studies assessed using the NOS (Table 3), three studies (60%) were rated as high quality (scores 7–9), and two (40%) as moderate quality (scores 5–6). The twelve case series studies assessed with the JBI checklist (Table 4) generally demonstrated clear case definitions and outcome reporting; however, limitations were noted in areas such as completeness of demographic reporting and appropriateness of statistical analysis in some studies. The extended quality assessment results based on the Newcastle–Ottawa Scale are shown in Tables 5, 6.

**TABLE 2:** Quality assessment of included studies with Cochrane Risk of Bias Tool (RoB 2).

Study (author, year)	Randomization process	Deviations from intended interventions	Missing outcome data	Measurement of the outcome	Selection of the reported result	Overall risk of bias
Ambiya et al. (27)	Some concerns	Low risk	Low risk	Low risk	Low risk	Some concerns
Sun et al. (15)	Some concerns	Low risk	Low risk	Low risk	Low risk	Some concerns
Roisman et al. (28)	Low risk	Low risk	Low risk	Low risk	Low risk	Low risk
Oh et al. (31)	Low risk	Low risk	Low risk	Some concerns	Low risk	Some concerns

The Cochrane Risk of Bias Tool (RoB 2) was used to assess the methodological quality of randomized controlled trials (RCTs). Each domain was judged as having a “Low risk,” “Some concerns,” or “High risk” of bias, and an overall judgment was derived accordingly. Randomization process: Assesses the appropriateness of random sequence generation and allocation concealment. Deviations from intended interventions: Considers knowledge of the intervention assignment, deviations from the intended protocol, and adherence to the intention-to-treat principle. Missing outcome data: Evaluates the amount and potential impact of missing data on the outcomes. Measurement of the outcome: Examines whether the outcome measurement could have been influenced by knowledge of the intervention received. Selection of the reported result: Assesses whether there was selective reporting from multiple analyses or outcome measurements.

**TABLE 3:** Quality Assessment of Included Studies with Newcastle–Ottawa Scale (NOS).

Study (author, year)	Selection (max 4)	Comparability (max 2)	Outcome (max 3)	Total score (/9)	Quality level
Torrellas et al. (14)	4	1	2	7	High
Uzlu et al. (16)	3	1	2	6	Moderate
Oribio-Quinto et al. (20)	4	1	2	7	High
Amoroso et al. (22)	4	0	3	7	High
Ambiya et al. (30)	3	1	2	6	Moderate

The Newcastle–Ottawa Scale (NOS) was used to assess the methodological quality of prospective cohort studies. The NOS evaluates studies across three domains: Selection (maximum 4 points), Comparability (maximum 2 points), and Outcome (maximum 3 points), with a total possible score of 9 points. Studies scoring 7–9, 5–6, and ≤ 4 points were considered to have high, moderate, and low methodological quality, respectively. Selection 1 (Representativeness of the exposed cohort): Were the cases representative of the defined population? Selection 2 (Selection of the unexposed cohort): Was the control group drawn from the same source population? Selection 3 (Ascertainment of exposure): Was exposure reliably ascertained? Selection 4 (Outcome not present at baseline): Was it demonstrated that the outcome of interest was not present at the start of the study? Comparability: Did the study control for the most important confounding factors in the design or analysis? Outcome 1 (Assessment of outcome): Were outcomes assessed objectively and reliably? Outcome 2 (Follow-up length): Was the follow-up period sufficiently long for the outcome to occur? Outcome 3 (Adequacy of follow-up): Was follow-up complete, with an acceptable loss-to-follow-up rate?.

**TABLE 4:** Quality assessment of included studies with JBI critical appraisal checklist.

Study (author, year)	Q1	Q2	Q3	Q4	Q5	Q6	Q7	Q8	Q9	Q10	“Yes” count
Işık et al. (17)	Y	Y	Y	Y	Y	Y	Y	Y	N/A	Y	9/9
Kayhan et al. (18)	Y	Y	Y	Y	U	Y	Y	Y	N/A	U	7/9
Liu et al. (19)	Y	Y	Y	Y	Y	Y	Y	Y	N/A	U	8/9
Gawêcki et al. (21)	Y	Y	Y	Y	Y	Y	Y	Y	N/A	U	8/9
Lanzetta et al. (29)	Y	Y	Y	Y	Y	Y	Y	Y	N/A	N	8/9
Kim et al. (23)	Y	Y	Y	Y	Y	Y	Y	Y	N/A	U	8/9
Kim et al. (24)	Y	Y	Y	Y	Y	Y	Y	Y	N/A	N	8/9
Maruko et al. (25)	Y	Y	Y	Y	Y	Y	Y	Y	N/A	U	8/9
Kustryn et al. (26)	Y	Y	Y	Y	Y	Y	Y	Y	N/A	U	8/9
Scholz et al. (35)	Y	Y	Y	Y	Y	Y	Y	Y	N/A	U	8/9
Yadav et al. (36)	Y	Y	Y	Y	Y	Y	Y	Y	N/A	N	8/9
Arsan et al. (37)	Y	Y	Y	Y	Y	Y	Y	Y	N/A	U	8/9

The Joanna Briggs Institute (JBI) Critical Appraisal Checklist for Case Series was used to assess the methodological quality of single-arm studies (case series). Each item was answered as “Y” (Yes), “N” (No), “U” (Unclear/Not Reported), or “N/A” (Not Applicable). The checklist does not prescribe a summary score threshold; a higher number of “Y” responses generally indicates higher reporting quality. The specific items are: Q1: Were there clear criteria for inclusion in the case series? Q2: Was the condition measured in a standard, reliable way for all participants? Q3: Were valid methods used for the identification of the condition for all participants? Q4: Did the case series have consecutive inclusion of participants? Q5: Did the case series have complete inclusion of participants? Q6: Was there clear reporting of the demographics of the participants? Q7: Was there clear reporting of the clinical information of the participants? Q8: Were the outcomes or follow-up results of cases clearly reported? Q9: Was there clear reporting of the presenting site(s)/clinic(s) demographic information? (Often marked N/A for single-center studies) Q10: Was statistical analysis appropriate?.

**TABLE 5:** Quality assessment of included studies with Newcastle–Ottawa Scale (NOS).

Study	Selection 1	Selection 2	Selection 3	Selection 4	Comparability	Outcome 1	Outcome 2	Outcome 3	Total (0–9 score)
Ambiya et al. (27)	1	1	0	1	0	1	1	0	5
Torrellas et al. (14)	1	1	0	1	1	1	1	0	6
Sun et al. (15)	1	0	1	1	1	1	0	1	6
Uzlu et al. (16)	1	1	1	0	1	1	0	1	6
Işık et al. (17)	1	1	1	0	1	1	0	1	6
Kayhan et al. (18)	1	1	1	0	0	1	1	0	5
Liu et al. (19)	1	1	1	0	2	1	0	1	7
Oribio-Quinto et al. (20)	1	1	1	1	1	1	0	1	7
Gawêcki et al. (21)	1	1	1	0	1	1	0	1	6
Roisman et al. (28)	1	1	1	0	1	1	1	0	6
Amoroso et al. (22)	1	1	1	1	0	1	1	1	7
Lanzetta et al. (29)	1	1	1	1	1	1	1	1	8
Ambiya et al. (30)	1	1	1	0	1	1	0	1	6
Kim et al. (23)	1	1	1	1	0	0	1	0	5
Kim et al. (24)	1	1	1	0	2	1	0	1	7
Maruko et al. (25)	1	1	1	1	0	1	0	1	6
Kustryn et al. (26)	1	1	1	0	0	1	0	1	5
Scholz et al. (37)	1	1	1	0	0	1	1	1	6
Ambiya et al. (27)	1	1	1	1	1	1	1	1	8
Torrellas et al. (14)	1	1	1	1	0	1	0	1	6
Sun et al. (15)	1	1	1	0	2	1	0	1	7

Selection 1, Is the exposed cohort representative?; Selection 2, Is the unexposed cohort selected from the same population?; Selection 3, Is the exposure factor accurately defined?; Selection 4, At baseline, were subjects confirmed free of the outcome of interest?; Comparability, Does the study design or analysis control for the most important confounding factors?; Outcome 1, Is the outcome assessment objective?; Outcome 2, Is the follow-up period sufficiently long to allow outcomes to occur?; Outcome 3, Is follow-up complete? Is the loss to follow-up rate acceptable?.

**TABLE 6:** GRADE evidence quality assessment of included studies.

Outcome measure	Number of studies/n	Sample size/n	Risk of bias	Inconsistency	Indirectness	Imprecision	Publication bias	Evidence level
BCVA	17	1,351	0	0	0	0	0	High
CMT	8	777	One level down	0	0	0	0	Moderate
CT	6	327	One level down	0	0	0	0	Moderate
CRT	8	489	0	0	0	0	0	High
SRFH	6	466	One level down	0	0	0	One level down	Low
ONL	2	106	0	0	0	One level down	One level down	Low

### 3.4 Outcome measurements

**FIGURE 2:**
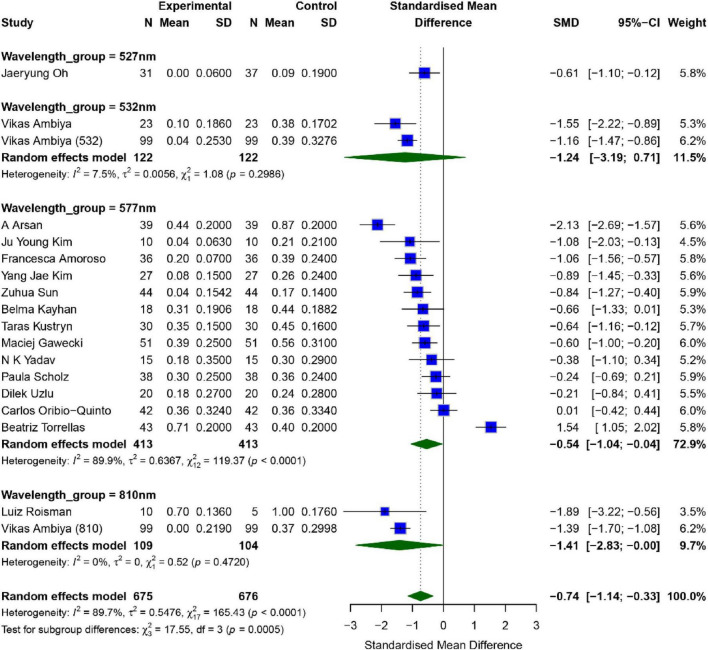
Meta-analysis of mean difference in BCVA after SML for chronic central serous chorioretinopathy.

A total of 18 studies assessed the impact of SML on patients’ Best Corrected Visual Acuity (BCVA), with the results shown in Figure 2. Overall analysis revealed significant heterogeneity across the studies (*I*^2^ = 89.7%, *P* < 0.01), therefore, a random-effects model was used for analysis. The combined effect size for the intervention and control groups was [*SMD* = −0.74, 95% *CI* (−1.14, −0.33), *P* = 0.001], indicating that the intervention group showed significantly better BCVA than the control group (*p* < 0.01).

To further explore the effects of different wavelengths of SML, we conducted a subgroup analysis. The results showed that in the two studies using a wavelength of 532 nm, the combined effect size was [*SMD* = −1.24, 95% *CI* (−3.19, 0.71), *P* = 0.078]; in the 14 studies using a wavelength of 577 nm, the combined effect size was [*SMD* = −0.54, 95% *CI* (−1.04, −0.04), *P* = 0.0371]; and in the two studies using a wavelength of 810 nm, the combined effect size was [*SMD* = −1.41, 95% *CI* (−2.83, −0.01), *P* = 0.049]. As only one study using a wavelength of 527 nm was included, a separate subgroup analysis for this group was not conducted. Significant improvements were observed for 577 nm and 810 nm, while the 532 nm group showed a positive trend without reaching statistical significance (*P* = 0.078). In terms of combined effect size, the improvement effect was most significant for the 810 nm wavelength. However, since only two studies were included, the interpretation of the results should be done with caution.

Publication bias analysis showed that neither the Egger test (*p* = 0.819) nor the Begg test (*p* = 0.5289) detected significant publication bias. However, sensitivity analysis and funnel plots revealed that the study by Beatriz Torrellas might be a potential source of publication bias (14). After excluding this study, the robustness of the remaining results was relatively strong (Supplementary Figures 1, 2).

#### 3.4.1 Central macular thickness

**FIGURE 3:**
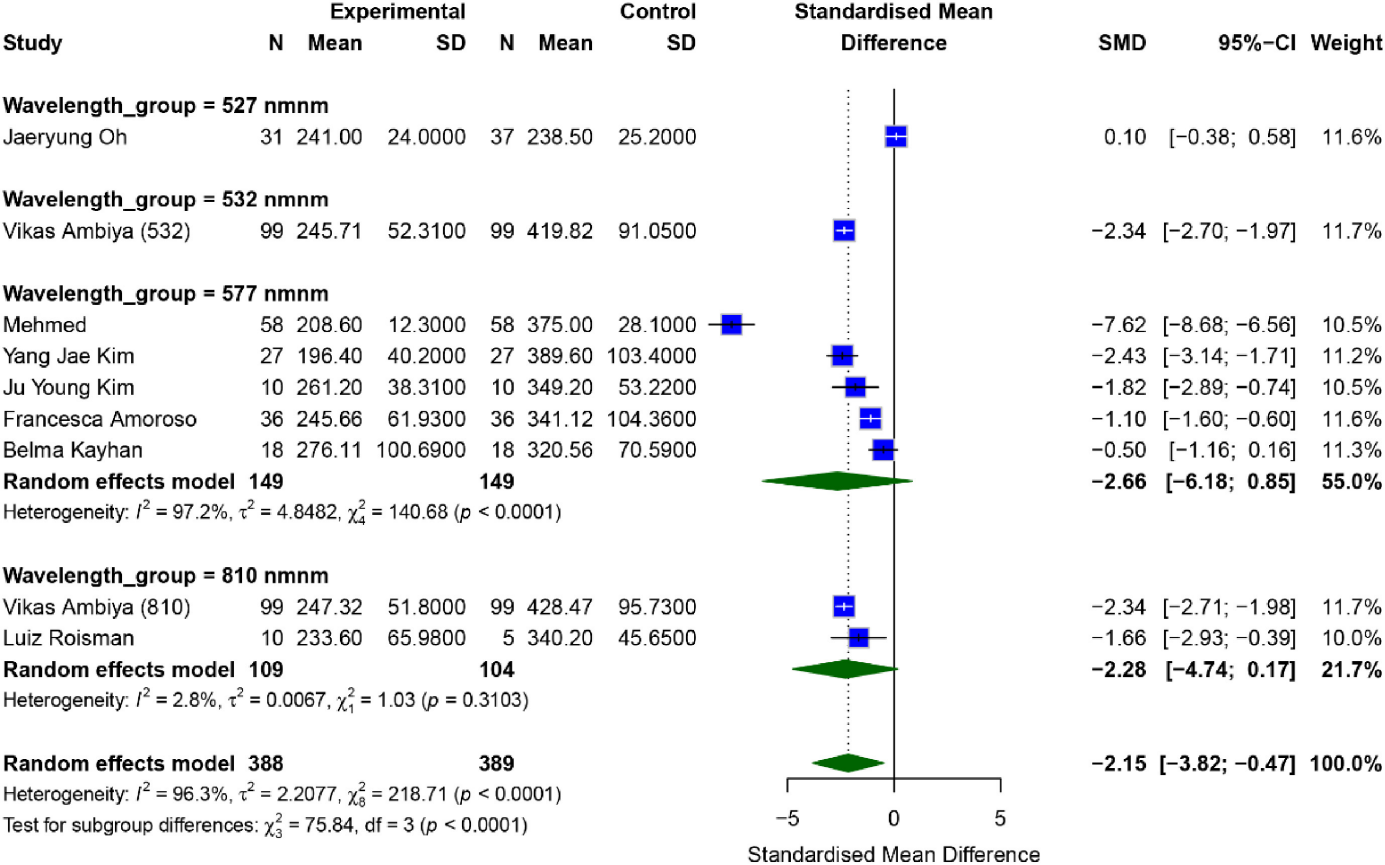
Meta-analysis of mean difference in CMT after SML for chronic central serous chorioretinopathy.

A total of eight studies assessed the effect of SML on patients’ Central Macular Thickness (CMT), with the results presented in Figure 3. Overall analysis revealed significant heterogeneity across the studies (*I*^2^ = 96.3%, *P* < 0.01); hence, a random-effects model was used for analysis. The combined effect size between the intervention and control groups was [*SMD* = −2.15, 95% *CI* (−3.82, −0.47), *P* = 0.018], indicating that the intervention group showed a significantly better CMT than the control group (*p* < 0.05).

To further investigate the effects of different wavelengths of SML, we performed a subgroup analysis. The results showed that in the five studies using a wavelength of 577 nm, the combined effect size was [*SMD* = −2.66, 95% *CI* (−6.18, 0.85), *P* = 0.103], while in the two studies using a wavelength of 810 nm, the combined effect size was [*SMD* = −2.28, 95% *CI* (−4.74, 0.17), *P* = 0.05]. Subgroup analysis indicated that while both 577 and 810 nm wavelengths showed a downward trend in CMT, the differences did not reach statistical significance (*P* > 0.05). The observed lack of significance in these subgroups might be attributed to the limited number of studies and high intra-group heterogeneity. Further research with larger sample sizes is required to confirm the wavelength-specific efficacy.

Publication bias analysis showed that the Egger test (*p* = 0.600) and the Begg test (*p* = 0.9170) did not reveal significant publication bias. However, sensitivity analysis and funnel plots indicated that the study by Mehmed Uğur Işık might be a potential source of publication bias (17). After excluding this study, the robustness of the remaining results was relatively strong (Supplementary Figures 3, 4).

#### 3.4.2 Choroidal thickness

**FIGURE 4:**
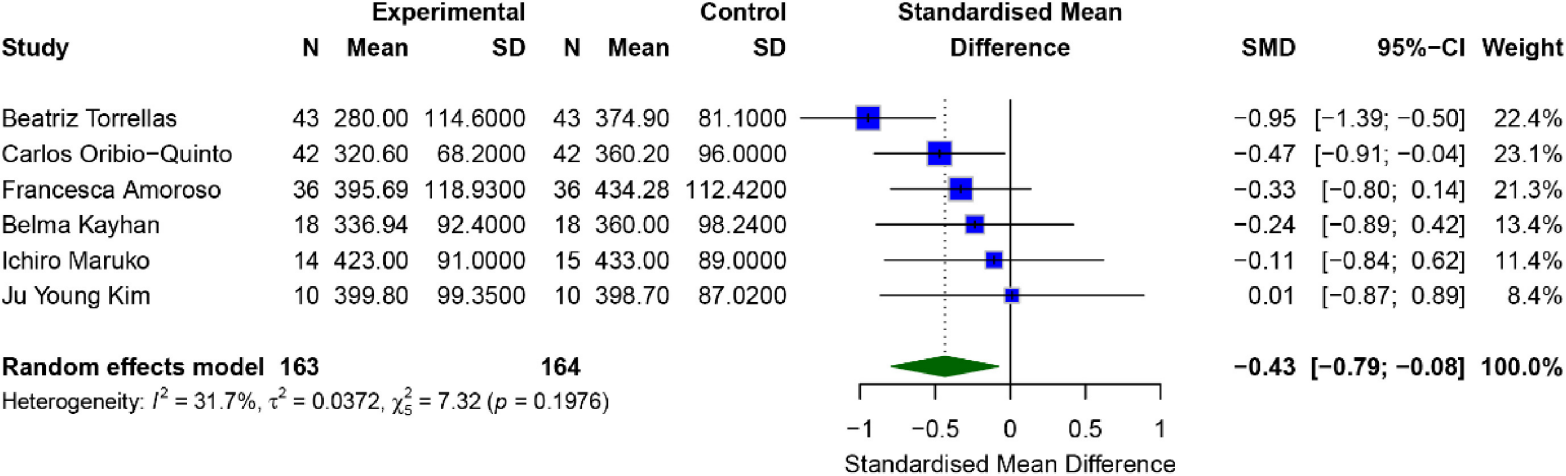
Meta-analysis of mean difference in CT after SML for chronic central serous chorioretinopathy.

A total of 6 studies evaluated the effect of SML on patients’ Choroidal Thickness (CT), all of which used a wavelength of 577 nm. The results are illustrated in Figure 4. There was moderate heterogeneity between the studies (*I*^2^ = 31.7%, *P* = 0.198), and therefore a fixed-effects model was employed for the analysis. The combined effect size for the intervention and control groups was [*SMD* = −0.43, 95% *CI* (−0.79, −0.08), *P* = 0.025], suggesting that the intervention group demonstrated a significantly greater improvement in CT compared to the control group (*p* < 0.05).

Publication bias analysis revealed that the Egger test (*p* = 0.130) did not indicate significant publication bias, whereas the Begg test (*p* = 0.0242) suggested the presence of publication bias. Nevertheless, sensitivity analysis and funnel plot inspection indicated that there was no major source of publication bias, and the robustness of the findings remained relatively strong (Supplementary Figures 5, 6).

#### 3.4.3 Central retinal thickness

**FIGURE 5:**
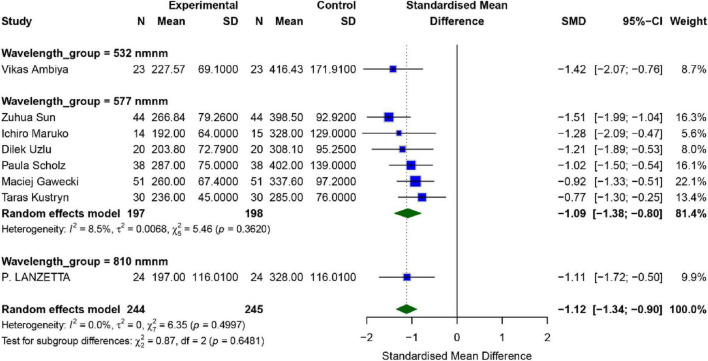
Meta-analysis of mean difference in CRT after SML for chronic central serous chorioretinopathy.

A total of 9 studies assessed the effect of SML on patients’ Central Retinal Thickness (CRT), with the results shown in Figure 5. The overall analysis revealed no observed heterogeneity between the studies (*I*^2^ = 0.0%, *P* = 0.499). The combined effect size for the intervention and control groups was [*SMD* = −1.12, 95% *CI* (−1.34, −0.90), *P* = 0.0001], indicating that the intervention group exhibited significantly better CRT compared to the control group (*p* < 0.01).

To further explore the effects of different wavelengths of SML, a subgroup analysis was conducted. The results showed that in the six studies using a wavelength of 577 nm, the combined effect size was [*SMD* = −1.09, 95% *CI* (−1.38, −0.80), *P* < 0.001]. Since only one study used a wavelength of 527 nm and another used 810 nm, separate subgroup analyses for these two groups were not performed.

Publication bias analysis revealed that neither the Egger test (*p* = 0.334) nor the Begg test (*p* = 0.3481) indicated significant publication bias. Sensitivity analysis and funnel plots showed that the robustness of the results was relatively strong (Supplementary Figures 7, 8).

#### 3.4.4 Height of subretinal fluid

**FIGURE 6:**
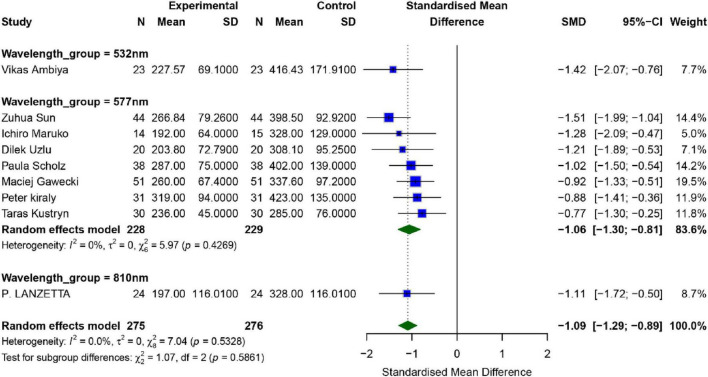
Meta-analysis of mean difference in SRFH after SML for chronic central serous chorioretinopathy.

A total of six studies evaluated the impact of SML on patients’ height of subretinal fluid (SRFH), as shown in Figure 6. Overall analysis revealed significant heterogeneity among the studies (*I*^2^ = 0.0%, *P* = 0.53), therefore, a random effects model was applied. The combined effect size between the intervention group and the control group was [*SMD* = −1.09, 95% *CI* (−1.29, −0.89), *P* < 0.001], indicating statistically significant difference in SRFH between the intervention group and the control group (*p* < 0.01).

To further explore the effects of different wavelengths of SML, subgroup analysis was performed. The results showed that in the five studies using a wavelength of 577 nm, the combined effect size was [*SMD* = −1.3, 95% *CI* (−1.3, −0.81), *P* < 0.001]. Since only one study used a wavelength of 527 nm, no separate subgroup analysis was conducted for this group.

Publication bias analysis showed significant publication bias in the studies, as indicated by the Egger test (*p* = 0.041), while the Begg test (*p* = 0.4524) did not reveal significant publication bias. Sensitivity analysis and funnel plots indicated that the study by Mehmed Uğur Işık could be the major source of potential publication bias (17). After excluding this study, the robustness of the remaining results was relatively strong (Supplementary Figures 9, 10).

#### 3.4.5 Outer nuclear layer

**FIGURE 7:**
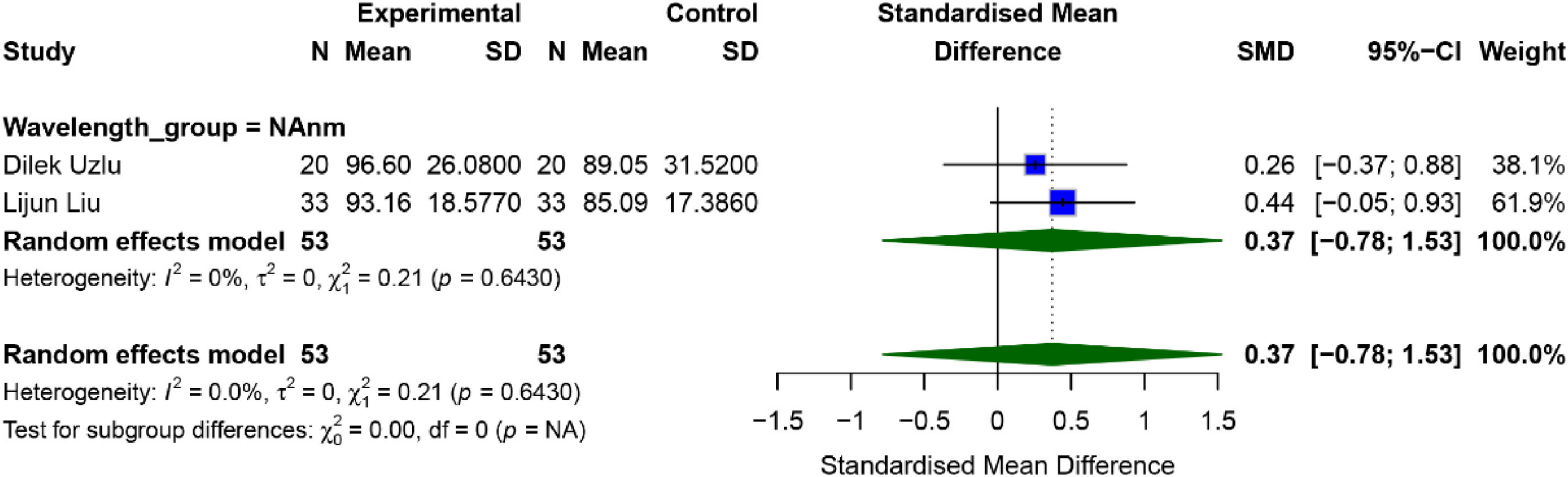
Meta-analysis of mean difference in ONL after SML for chronic central serous chorioretinopathy.

Two studies evaluated the effects of SML on the Outer Nuclear Layer (ONL), both utilizing the 577 nm wavelength. Results are presented in Figure 7. No significant heterogeneity was observed across studies (*I*^2^ = 0%, *P* = 0.643). The pooled effect size between the intervention and control groups was [*SMD* = 0.37, 95% *CI* (−0.78, 1.53), *P* = 0.153], indicating no statistically significant difference in ONL outcomes (*p* > 0.05). Due to the limited number of studies, funnel plot analysis, sensitivity analysis, Egger’s test, and Begg’s test were not conducted.

#### 3.4.6 Safety

Among the 19 studies evaluating adverse events following SML treatment, 5 studies reported adverse reactions in cCSC patients’ post-treatment (Table 1). Specifically, Zuhua Sun et al. found that 12% of patients in the SML group exhibited mild RPE depigmentation (15). Oribio-Quinto et al. reported one case of vision loss associated with progressive outer retinal layers (ORL) and RPE atrophy, manifested by a 15-character decrease in letter recognition ability (20). Ichiro Maruko et al. recorded one case of RPE damage (25). Taras Kustryn et al. reported five cases of localized RPE scarring (26). Additionally, Oh et al. reported five cases of ocular adverse events (31). These findings suggest that while SML treatment generally demonstrates a high safety profile in cCSC patients, attention should be paid to the potential for RPE-related adverse reactions.

### 3.5 Evidence quality assessment

Concluded that subthreshold micropulse laser treatment has a beneficial effect on chronic central serous chorioretinopathy. The GRADE evidence quality assessments for the included studies are shown in Table 6. BCVA and CRT were rated as high quality, CMT and CT as moderate quality, and ONL and SRFH as low quality. The current quality of evidence is limited (with GRADE ratings mostly low to moderate), and large-scale randomized controlled trials are still needed for validation, which would enhance scientific rigor.

## 4 Discussion

Chronic CSC carries a risk of irreversible photoreceptor damage and permanent vision loss if not treated in a timely manner (32). Conventional laser therapy can promote subretinal fluid absorption but is associated with the risk of RPE and photoreceptor injury, whereas photodynamic therapy is effective but costly and may lead to complications such as choroidal ischemia and RPE alterations. These limitations highlight the need for safer and more targeted therapeutic approaches. Subthreshold micropulse laser has emerged as a promising alternative because it delivers therapeutic effects below the threshold of visible tissue damage (33). It minimizes iatrogenic injury to the RPE and photoreceptors, does not require a photosensitizer, and therefore avoids PDT-related complications, while also allowing repeatable treatment (32). Although the clinical value of SML in cCSC is increasingly recognized, its efficacy and safety still need to be further validated by large-scale, high-quality clinical trials.

This study comprehensively evaluated the clinical efficacy of SML therapy in the treatment of cCSC using systematic review and meta-analysis methods, demonstrating significant improvements in key clinical indicators. A related systematic review published in 2025 also evaluated SML therapy for CSC (32, 34). Differences in the number of included studies between that review and the present analysis are mainly attributable to differences in eligibility criteria and study scope. Our review specifically focused on chronic CSC and predefined quantitative anatomical outcomes, and we applied stricter inclusion criteria and structured quality grading. In addition, we performed wavelength-specific subgroup meta-analyses and comprehensive bias and certainty assessments. Therefore, the present study provides a more granular and methodologically stratified evaluation of SML efficacy and safety in Ccsc (8, 9, 34). Specifically, SML intervention significantly enhanced BCVA versus the control group, suggesting wavelength-independent efficacy in visual acuity improvement and providing critical evidence for clinical parameters selection. Regarding retinal morphological indicators, SML also showed significant therapeutic effects, as evidenced by improvements in key indicators such as CMT, CT, and CRT. These findings confirmed its role in promoting the absorption of subretinal fluid and alleviating pathological state in the retina and choroid, with particularly notable effects observed at 577 nm. These findings align with the observations reported by Su Zhang et al., demonstrating that post-treatment CMT was significantly reduced in the 577 nm SML cohort, with SRF absorption rates compared to conventional laser therapy. Although SRFH improvement after SML was not statistically significant, most studies indicated a positive trend, possibly influenced by sample size and follow-up duration (22, 23).

Research on SML with wavelengths of 810, 577, 532, and 527 nm was systematically included, among which the 577 nm wavelength exhibited the widest clinical application and received the most extensive investigation. Significant differences existed among wavelengths regarding tissue penetration depth, energy absorption efficiency, and selective effects on RPE cells. From a mechanistic perspective, the effects of SML on the retina and choroid extend beyond selective melanosome heating (8, 9). Subthreshold micropulse stimulation provides low-duty-cycle thermal stress to RPE cells without coagulative damage, activating cellular stress-response pathways and improving RPE metabolic and fluid transport function (8). This functional restoration of RPE may enhance subretinal fluid resorption and help re-establish the outer blood–retinal barrier in Ccsc (35, 36). RPE activation may also indirectly modulate choroidal circulation through RPE–choroid signaling, which could explain the choroidal thickness reductions observed in several studies. Differences between wavelengths are likely related to absorption and penetration properties (27). Shorter wavelengths (532–577 nm) are more strongly absorbed by melanin and hemoglobin, providing greater RPE selectivity and more localized effects, whereas longer wavelengths (810 nm) penetrate deeper and may induce broader sub-RPE and choroidal bio-stimulation. However, wavelength-related differences should be interpreted cautiously and confirmed in direct comparative trials (8, 34). Results indicated that 810 nm SML yields superior improvement in BCVA, while 577 nm SML exceled at enhancing retinal morphological indicators such as foveal retinal thickness and subretinal fluid absorption. However, due to limited study numbers and high heterogeneity, no consensus existed on optimal wavelength for efficacy and safety in treating cCSC. Moreover, wavelength selection may be influenced by patient-specific factors (e.g., RPE pigment distribution, choroidal thickness), lesion location (fovea or parafovea), and disease severity, significantly complicating treatment parameters optimization. Existing evidence suggests that the 577 nm wavelength, due to its high pigment absorption efficiency and precise RPE targeting, is more suitable for patients with superficial lesions primarily involving RPE dysfunction, especially when treating near the fovea, where it demonstrates higher safety (34, 37). Leveraging large-scale computational models, researchers including Ivanova et al. established RPE-targeted micro-pulse parameters, with efficiency-selectivity metrics quantifying tissue damage, providing a theoretical foundation for personalized SML therapy (38).

High heterogeneity was observed in some pooled outcomes (such as BCVA, CMT, and SRFH; I^2^ > 80%), which should be considered when interpreting the pooled estimates. Several factors may explain this variability. The included studies differed in design and comparator choice, including observation-only controls, conventional threshold laser, and photodynamic therapy, which may influence the magnitude of comparative effects. Baseline characteristics also varied considerably, including disease duration, prior treatments, and recurrence status. In addition, treatment parameters were not uniform across studies, with differences in wavelength, duty cycle, spot size, energy settings, and exposure duration. Follow-up periods ranged from short-term to longer-term assessments, potentially contributing to outcome discrepancies. Methodological quality also varied, as both randomized and non-randomized studies were included, and some lacked masking or control groups. Geographic and population differences may have further contributed to variability. Subgroup and sensitivity analyses reduced heterogeneity after exclusion of outlier studies, supporting the overall directional robustness of the main findings despite residual clinical and methodological heterogeneity.

In terms of safety, existing studies suggest that inappropriate SML parameter settings may induce retinal damage or retinal stress responses (15). Among the studies included in this meta-analysis, a minority reported treatment-related adverse events, mainly including RPE depigmentation (26), RPE atrophy (25), subretinal fluid persistence, and localized RPE scarring (20). Most reported events were mild and did not result in severe visual impairment or require additional intervention. Rare but more serious cases have been described, such as vision loss associated with progressive outer retinal layer and RPE atrophy (39), although a direct causal relationship remains uncertain. Overall, SML appears to have a favorable safety profile in cCSC compared with conventional threshold laser or PDT; however, parameter selection remains critical. It should also be noted that follow-up durations were relatively short in many included studies and adverse event reporting was not fully consistent across reports, which may lead to underestimation of long-term or rare safety outcomes. Therefore, individualized parameter titration and longer-term structural and functional monitoring are recommended in clinical practice.

Several limitations of this meta-analysis should be acknowledged. First, substantial heterogeneity was observed in some pooled outcomes, which may be related to differences in study design, patient characteristics, prior treatments, and SML parameter settings across studies. Second, a considerable proportion of the included studies were non-randomized or single-arm designs, which may introduce selection bias and limit the overall certainty of evidence despite formal quality assessment and GRADE evaluation. Third, follow-up durations in many studies were relatively short, which restricts assessment of long-term anatomical and functional outcomes. In addition, treatment protocols and reporting standards were not fully uniform across studies, which may have contributed to residual variability that could not be completely addressed by subgroup and sensitivity analyses. Finally, the literature search was completed in January 2025; therefore, more recently published studies were not included. Future updated and prospectively registered meta-analyses incorporating newly emerging evidence will be important to further strengthen the conclusions.

## 5 Conclusion

This systematic review and meta-analysis comprehensively evaluated the efficacy and safety of SML in the treating cCSC, consolidating existing evidence to support clinical decision-making. Subgroup analysis highlighted wavelength-specific effects, offering the development of personalized treatment strategies. Future research should aim to standardize treatment parameters, optimize intervention timing, and incorporate long-term follow-up to refine the clinical application of SML therapy in cCSC.

## Data Availability

The original contributions presented in this study are included in the article/Supplementary material, further inquiries can be directed to this corresponding author.
