# Supplementary material for: Efficacy and safety of subthreshold micropulse laser for chronic central serous chorioretinopathy: a systematic review and meta-analysis
Source: Front Med (Lausanne). 2026 Mar 13;13:1785787. doi: 10.3389/fmed.2026.1785787 (PMC13023777; doi:10.3389/fmed.2026.1785787)
Supplement: Supplementary file 1 [file Table_1.docx]

**Supporting information**

**Figure S1. Sensitivity analysis of the meta-analysis on the effect of micropulse laser therapy on chronic central serous chorioretinopathy based on BCVA.**

**
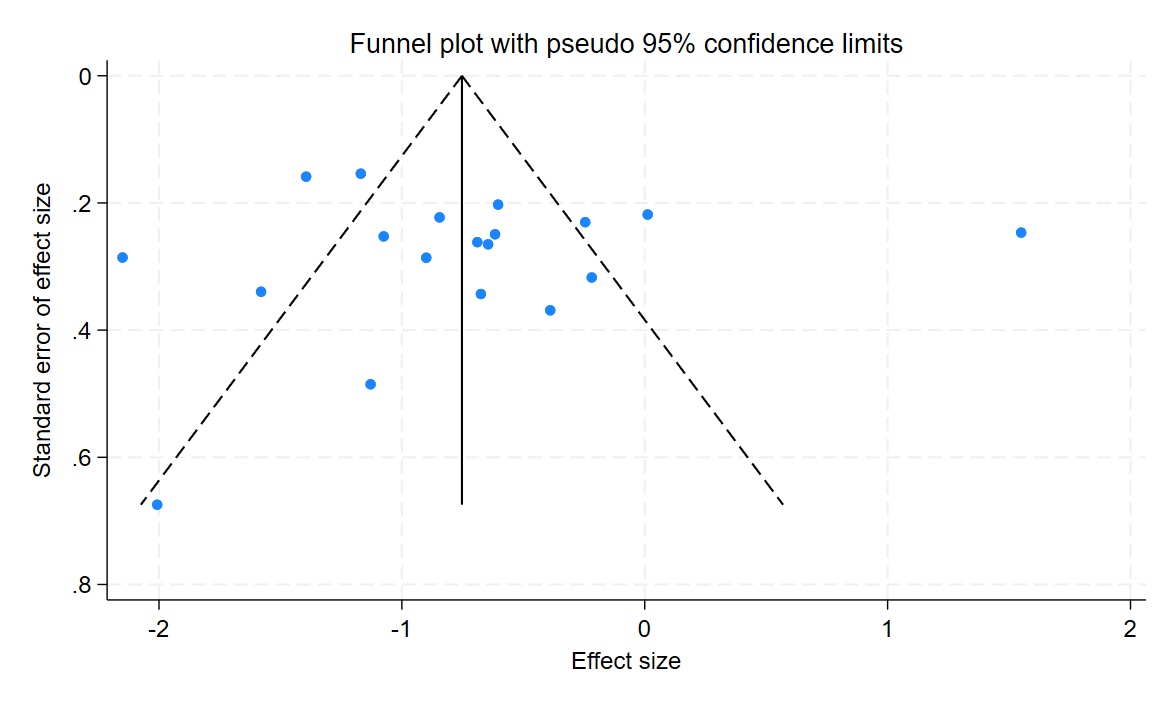
**

**Figure S2.** **Funnel plot analysis of the effect of micropulse laser therapy on BCVA in chronic central serous chorioretinopathy patients.**

**Figure S3.** **Sensitivity analysis of the meta-analysis on the effect of micropulse laser therapy on chronic central serous chorioretinopathy based on CMT.**

**
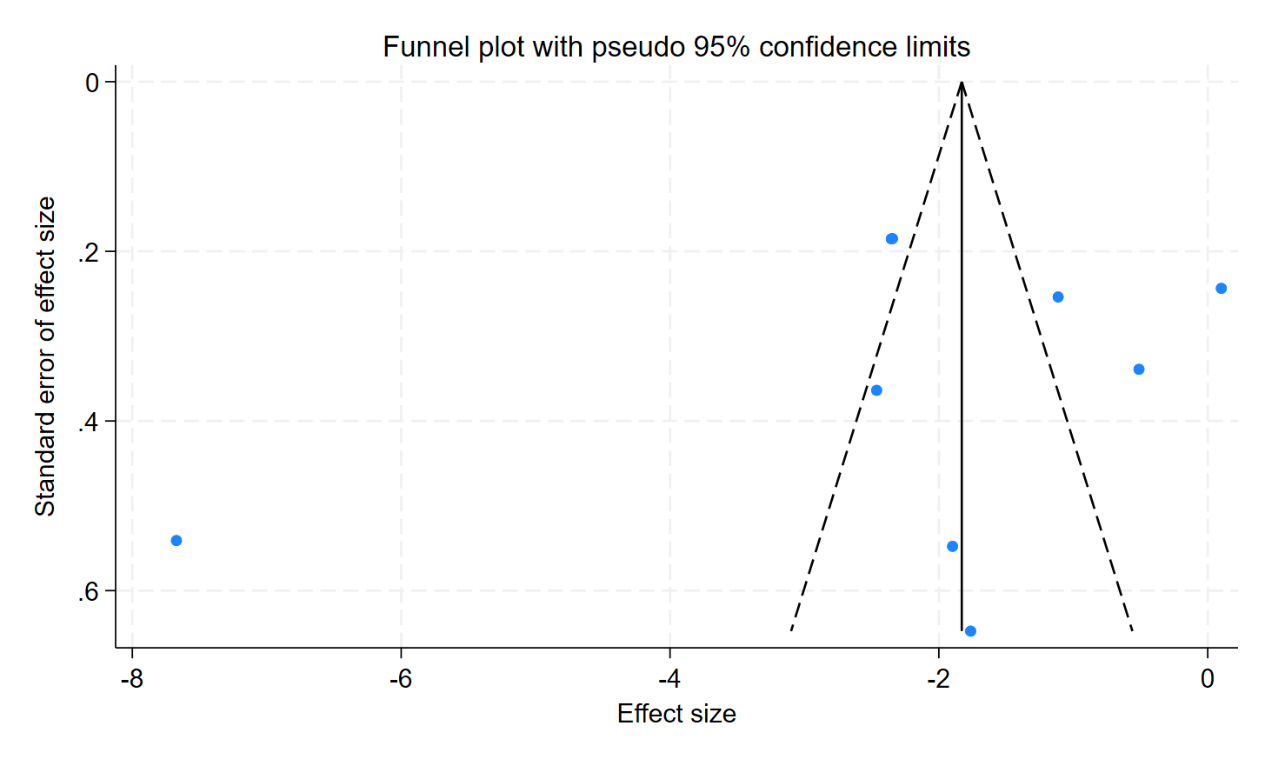
**

**Figure S4.** **Funnel plot analysis of the effect of micropulse laser therapy on CMT in chronic central serous chorioretinopathy patients.**

**Figure S5.** **Sensitivity analysis of the meta-analysis on the effect of micropulse laser therapy on chronic central serous chorioretinopathy based on CT.**

**
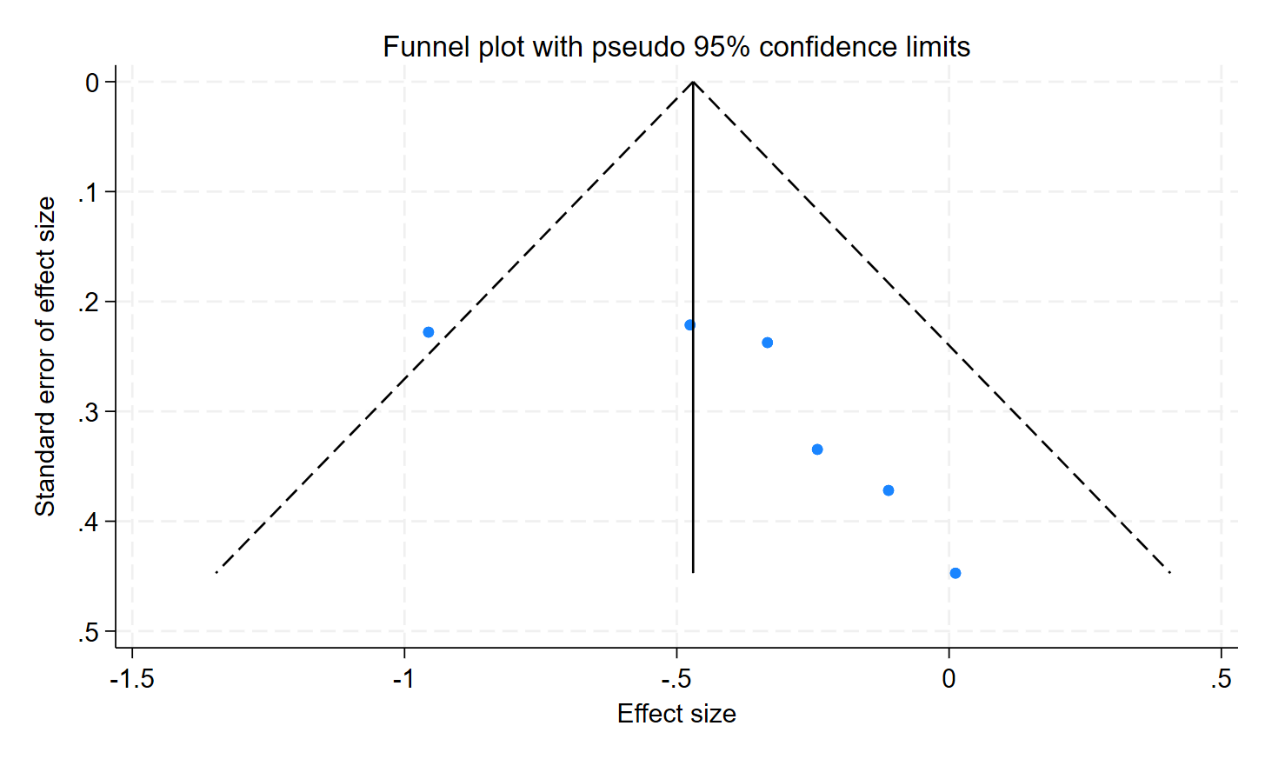
**

**Figure S6.** **Funnel plot analysis of the effect of micropulse laser therapy on CT in chronic central serous chorioretinopathy patients.**

**Figure S7.** **Sensitivity analysis of the meta-analysis on the effect of micropulse laser therapy on chronic central serous chorioretinopathy based on CRT.**

**
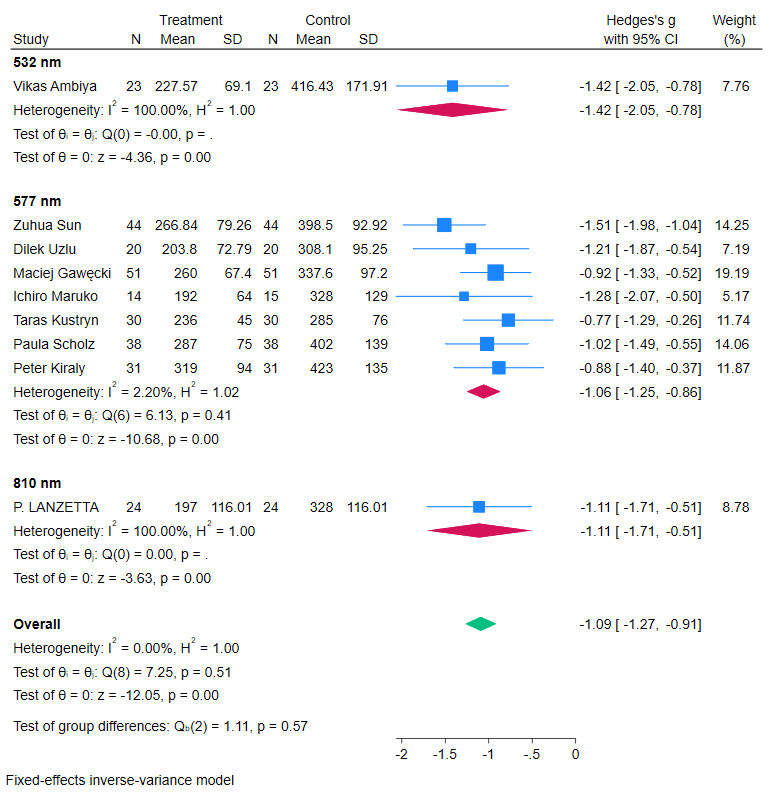
**

**Figure S8.** **Funnel plot analysis of the effect of micropulse laser therapy on CRT in chronic central serous chorioretinopathy patients.**

**Figure S9. Sensitivity analysis of the meta-analysis on the effect of micropulse laser therapy on chronic central serous chorioretinopathy based on SRFH.**

**
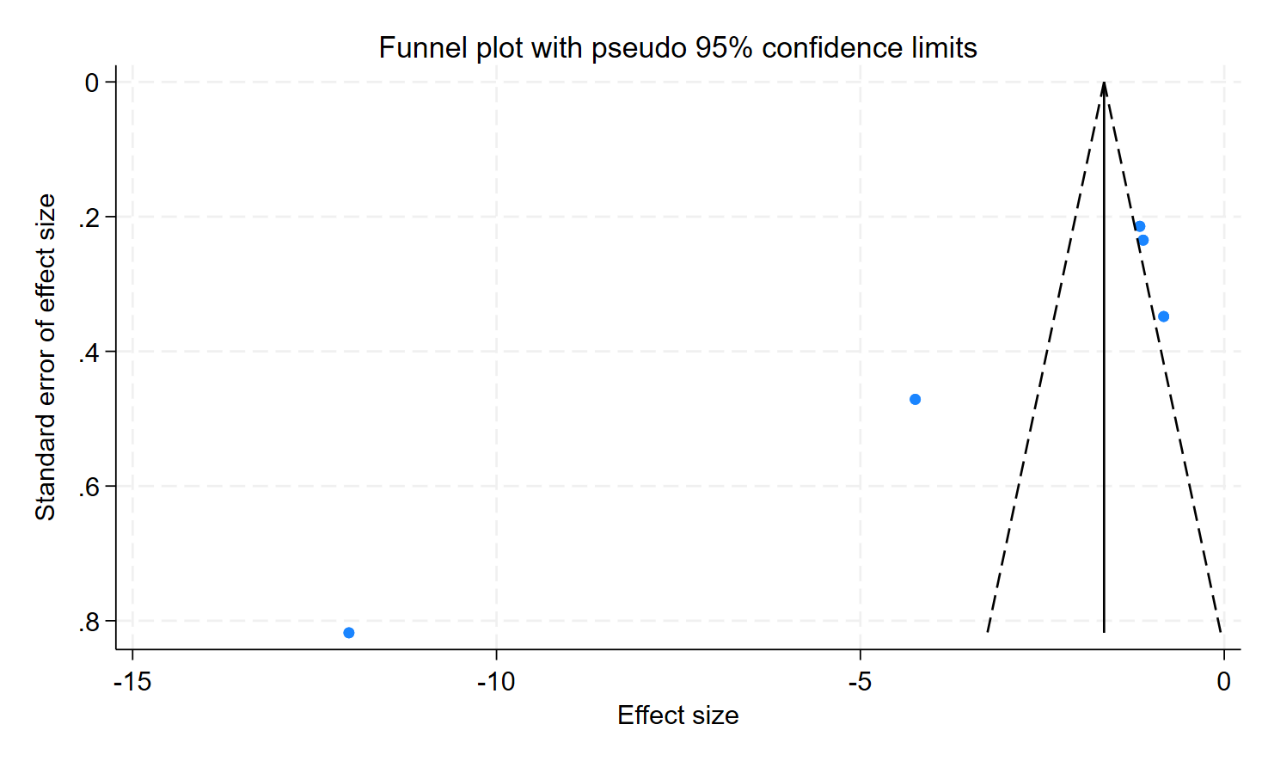
**

**Figure S10. Funnel plot analysis of the effect of micropulse laser therapy on SRFH in chronic central serous chorioretinopathy patients.**

***Supplementary Table 1. Search strategy in PubMed, Embase, Web of Science and Cochrane Library***

| **Set** | **PubMed** | **Search type** | **Results** |
| --- | --- | --- | --- |
| #1 | ("chronic central serous chorioretinopathy"[tiab] OR "chronic csr"[tiab] OR "chronic cscr"[tiab] OR "persistent central serous chorioretinopathy"[tiab]) AND ("subthreshold micropulse laser"[tiab] OR "micropulse laser"[tiab] OR "subthreshold laser"[tiab] OR "sml"[tiab] OR "mpl"[tiab]) | Advanced | 102 |
| **Set** | **Embase** | **Search type** | **Results** |
| #1 | 'chronic central serous chorioretinopathy'/exp OR 'chronic central serous chorioretinopathy' OR (chronic AND ('central'/exp OR central) AND serous AND ('chorioretinopathy'/exp OR chorioretinopathy)) | Advanced | 1337 |
| #2 | 'subthreshold micropulse laser therapy'/exp OR 'subthreshold micropulse laser therapy' OR (subthreshold AND ('micropulse'/exp OR micropulse) AND ('laser'/exp OR laser) AND ('therapy'/exp OR therapy)) | Advanced | 366 |
| #3 | #1 AND #2 | Advanced | 101 |
| **Set** | **Web of Science** | **Search type** | **Results** |
| #1 | TS=("central serous chorioretinopathy" OR "central serous retinopathy" OR CSC OR CSCR)  AND  TS=(chronic OR persistent OR recurrent OR "long-standing" OR nonresolving)  AND  TS=(micropulse OR "micro-pulse" OR subthreshold OR "sub-threshold" OR  "subthreshold micropulse" OR "micropulse laser" OR "micropulse diode" OR "577 nm" OR "810 nm" OR "yellow laser") | Advanced | 152 |
| **Set** | **Cochrane Library** | **Search type** | **Results** |
| #1 | ( ("chronic central serous chorioretinopathy" OR ("central serous chorioretinopathy" AND (chronic OR persistent OR recurrent))):ti,ab,kw AND ("subthreshold micropulse laser therapy" OR "subthreshold micropulse laser" OR (subthreshold NEXT micropulse NEXT laser)):ti,ab,kw ) | Advanced | 28 |
